# Risk Assessment of Etanercept in Mice Chronically Infected With *Toxoplasma gondii*

**DOI:** 10.3389/fmicb.2018.02822

**Published:** 2018-11-21

**Authors:** Jing Yang, Luyao Wang, Dongmei Xu, Ding Tang, Senyang Li, Fen Du, Lixia Wang, Junlong Zhao, Rui Fang

**Affiliations:** ^1^State Key Laboratory of Agricultural Microbiology, College of Veterinary Medicine, Huazhong Agricultural University, Wuhan, China; ^2^Hubei Centre for Animal Diseases Control and Prevention, Wuhan, China; ^3^Hubei Provincial Centre for Diseases Control and Prevention, Wuhan, China

**Keywords:** *Toxoplasma gondii*, tumor necrosis factor, etanercept, chronic infection, brain cysts reactivation

## Abstract

*Toxoplasma gondii* (*T. gondii*) is a zoonotic parasite that severely harms the health of the host. The cysts of *T. gondii* can reactivate from bradyzoites to tachyzoites, if the individual develops low or defective immunity, causing lethal toxoplasmosis. The host resists *T. gondii* infection by mediating Th1-type cellular immunity to generate pro-inflammatory cytokines. Tumor necrosis factor (TNF) is an important pro-inflammatory cytokine, which can induce lysosomal fusion of parasitophorous vacuole (PV) to kill parasites. Etanercept is a soluble TNF receptor fusion protein, which is widely used clinically to cure autoimmune diseases. The effects and specific molecular mechanisms of etanercept treatment on patients co-infected with autoimmune diseases and chronic toxoplasmosis are rarely reported. In our study, a mouse model of chronic infection with *T. gondii* and murine macrophages RAW264.7 cells infected with *T. gondii* were employed to investigate the impact of etanercept on the status of chronic infection. The cytokines levels and a series of phenotypic experiments *in vivo* and *in vitro* were measured. In the present study, the expression levels of TNF, IL-1β, and IL-6 were decreased and the brain cysts number was increased in mice chronically infected with *T. gondii* after being treated with etanercept. *In vivo* experiments confirmed that etanercept caused a decrease in the immune levels of the mice and activated the brain cysts, which would lead to conversion from chronic infection to acute infection, causing severe clinical and pathological symptoms. Murine macrophages RAW264.7 cells were pretreated with etanercept, and then infected with *T. gondii*. *In vitro* experiments, the expression levels of cytokines were decreased, indicating that etanercept could also reduce the cells’ immunity and promote the transformation of bradyzoites to tachyzoites, but did not affect the intracellular replication of tachyzoites. In summary, etanercept treatment could activate the conversion of bradyzoites to tachyzoites through reducing host immunity *in vivo* and *in vitro*. The results obtained from this study suggest that the use of etanercept in patients co-infected with autoimmune diseases and chronic toxoplasmosis may lead to the risk of activation of chronic infection, resulting in severe acute toxoplasmosis.

## Introduction

*Toxoplasma gondii* (*T. gondii*) is an important zoonotic parasite that can infect all warm-blood animals, including humans (Nicolle and Manceaux, 2009). Almost one third people worldwide are infected with this intracellular parasite, and toxoplasmosis has become a global, serious problem (Halonen and Weiss, 2013). Clinical symptoms related to toxoplasmosis included abortion, ocular disease, and encephalitis (Pappas et al., 2009; Dubey et al., 2012). Tachyzoites can differentiate into slow-growing cysts after activating host immune responses then showing no clinical symptom in immunocompetent host (Mercier et al., 2011). However, for some host whose immunity is inhibited, such as HIV-infected or transplanted patients, the cysts can reactivate from bradyzoites to tachyzoites, and then cause lethal toxoplasmosis (Giese et al., 2004; Miedema et al., 2013).

Tumor necrosis factor (TNF), a pro-inflammatory cytokine is secreted by activated macrophages and Th1-type cells, and is involved in the host’s immune responses against parasites (Munoz-Carrillo et al., 2017; Degbe et al., 2018). The host resists *T. gondii* infection by mediating Th1-type cellular immunity to generate pro-inflammatory cytokines. The role of TNF during parasites infection greatly depends on the strain of parasites, the state of infection, and the amount of induced TNF (El-Sayed et al., 2016). The number of *T. gondii* in macrophages reduces through lysosomal fusion and parasitophorous vacuole (PV) disruption induced by TNF (Andrade et al., 2006). It has been reported that the use of anti-TNF antibodies against mice or macrophages can induce the transformation of bradyzoites to tachyzoites and tachyzoites proliferate (Young and McGwire, 2005). TNF is used as a costimulatory molecule and plays an important role in the stimulation of IFN-γ production in NK cells. Additionally, IFN-γ is produced only when the *T. gondii* antigen and TNF co-stimulate NK cells (Langermans et al., 1992; Pittman and Knoll, 2015). Etanercept is a soluble TNF receptor fusion protein with a long half-life, which is widely used clinically (Weinblatt et al., 1999; Almon et al., 2017). The main action mechanism of etanercept is to directly bind to TNF, reducing its biological effectiveness, thus inhibiting autoimmune responses and inflammatory responses mediated by TNF (Kikuchi et al., 2012). Etanercept is widely applied to treat autoimmune diseases such as rheumatoid arthritis, ankylosing spondylitis, and psoriasis (Zou et al., 2002; Yurttutan et al., 2014). Recent research has demonstrated that etanercept can reduce inflammation and lethality in mice infected with Japanese encephalitis virus (Ye et al., 2014).

However, the effects and specific molecular mechanisms of etanercept treatment on patients co-infected with autoimmune diseases and chronic toxoplasmosis are rarely reported. Therefore, whether etanercept causing an increased infection risk is a key question needed to be concerned in host co-infected with autoimmune diseases and toxoplasmosis. In the present study, we explored the possible effects of etanercept on latent toxoplasmosis *in vivo* and *in vitro*, and illustrated that etanercept could reactive bradyzoites to tachyzoites through reduction of host immunity ability, leading to lethal acute infection. The results obtained from this study may provide reference for drug therapy in clinical patients co-infected with autoimmune diseases and chronic toxoplasmosis.

## Materials and Methods

### Animals and Parasites

Ten weeks male BALB/c mice were obtained from the Hubei Provincial Centre for Diseases Control and Prevention (Wuhan, China). All mice were housed under specific pathogen-free conditions at 25°C under a 12 h lightdark cycle in the Hubei Provincial Centre for Disease Control and Prevention (Wuhan, China). *T. gondii* type II ME49 strain was maintained in BALB/c mice in the form of tissue cysts. To establish chronic infection in naive mice, these mice were randomly divided into 7 groups (*n* = 6 in each group), and each mouse was orally infected with 10 cysts of ME49 strain in 100 μL phosphate-buffered saline (PBS) counted by hemocytometer simultaneously. Serum of mice were collected from infected mice to detect the bradyzoite antigen 1 (BAG1) levels after 4 weeks post-infection. The anti-BAG1 positive mice were used as chronically infected ones for further experiments.

### Etanercept Administration to Mice Chronic Infection With *T. gondii*

Etanercept was purchased from Pfizer Pharmaceuticals Limited (New York, NY, United States). As a recombinant human TNFR2-IgFc fusion protein, etanercept is reported to effectively bind and neutralize mouse TNF (Yoshitaka et al., 2014). The anti-BAG1 positive mice were randomly divided into two groups: *T. gondii*-infected group (ME49; *n* = 10); and *T. gondii*-infected and etanercept-treated group (ME49+Etan; *n* = 10). Two other uninfected mice served as controls: PBS-treated group (PBS; *n* = 10); only etanercept-injected group (Etan; *n* = 10). Etanercept was intraperitoneally administered to mice of ME49+Etan group at a dose of 1 mg/kg/week for 4 weeks (El-Sayed et al., 2016). Mice in the PBS group received PBS and mice in the Etan group received etanercept.

All mice were monitored daily to assess behavior and mortality, behavioral scoring was applied to evaluate clinical symptoms (Mishra and Basu, 2008). After 4 weeks post-infection, all the mice were euthanized by inhalation of CO_2_. The brain tissues and serum were collected for subsequent experiments, the brain were homogenized to measure the number of Toxoplasma cysts by DBA-FITC staining, as described previously (Buchholz et al., 2013). All experiments were carried out in accordance with guidelines from the Laboratory Animal Research Centre of Hubei province. This protocol was approved by the Ethical Committee on Animal Research at Huazhong Agricultural University (HZAUMO-2016-025), and all efforts were made to minimize the suffering of animals.

### Histological Analysis

Brain tissues were acquired and fixed with 4% paraformaldehyde for 24 h. After dehydration and transparention, the brain tissues were stained with haematoxylin and eosin (H&E) using standard histological techniques, then the histopathological changes were observed with a light microscopy (Olympus, Japan). IPP6.0 software was used to quantitative count the number of inflammatory infiltrating cells from the same area around the blood vessels from three independent pathological sections. The method of counting inflammatory infiltrating cells in tissues was performed as described previously (Horai et al., 2017). Briefly, the nucleus and inflammatory infiltrating cells could be dyed blue after H&E staining. Thus, the area inside the profile of nucleus was filled to be set as one object through adding measurement conditions with fill holes. Subsequently, inflammatory cells could be distinguished from nucleus by setting the size conditions and inflammatory infiltrating cells could be analyzed quantitatively.

### Bradyzoite Differentiation *in vitro*

The method of inducing bradyzoite differentiation *in vitro* was performed as described previously (Xia et al., 2018). Briefly, ME49 stain tachyzoites were forced to egress by needle passages and allowed to invade HFF monolayers seeded in T25 flasks. After 1 h under standard growth conditions, the non-invaded extracellular parasites were washed off, then the parasites were sequentially cultured in T25 flasks under bradyzoite-inducing conditions (RPMI 1640 medium supplemented with 50 mM HEPES and 1% fetal bovine serum, PH 8.2, ambient CO_2_) for 4 days.

To verify that the parasites were indeed bradyzoites, HFF monolayers were seeded on coverslips in 24-well plates, and then ME49 strain tachyzoites were used to invade HFF monolayers (parasites/well). After 1 h under standard growth conditions, non-invaded parasites were washed away with PBS and the invaded ones were grown in differentiation medium with ambient CO_2_ for 4 days. Subsequently, the cells were fixed with 4% paraformaldehyde, and permeabilized with 0.1% Triton X-100 for 15 min. The cells were stained with rabbit anti-TgALD and mouse anti-TgBAG1 as described previously (Shen and Sibley, 2014; Yang J. et al., 2017; Xia et al., 2018). Then primary antibodies were detected by Alexa Fluor 594-conjugated goat anti-rabbit IgG (Life Technologies, Inc., Rockville, MD, United States) and Alexa Fluor 488-conjugated goat anti-mouse IgG (green, for bradyzoites) second antibodies. After staining, the bradyzoites differentiation was determined using an Olympus BX53 fluorescence microscope (100× with NA = 1.4) equipped with a Zeiss Axiocam 503 mono camera. The images were stored using Zeiss Zen control software on a personal computer.

### Cell Culture and Treatment

RAW264.7 cells were acquired from the American Type Culture Collection (ATCC TIB-71^TM^). The cells were cultured in DMEM supplemented with 10% FBS and were incubated continually at 37°C with 5% CO_2_. The cells were pretreated with etanercept (10, 100, 1000 ng/mL) for 1h and then infected with bradyzoites. Cells treated with PBS served as a blank control group, and cells only treated with etanercept (10, 100, 1000 ng/mL) served as a drug control group.

### Cell Viability Assay

RAW264.7 cells were plated at a density of 1 × 10^5^ cells/mL in 96-well plates at 37°C with 5% CO_2_ for 12 h and then the cells were treated with etanercept at the dose of 10, 100, or 1000 ng/mL. After 24 h incubating, 20 μL MTT was added into 96-well plates on the shaker table for 10 min, and the optical density (*OD*) value was determined at 490 nm using a microplate reader.

### ELISA Assay

The effects of etanercept on the expression of cytokines were detected in serum and cells, the proteins expression levels of TNF, IL-1β, and IL-6 were determined using enzyme-linked immunosorbent assay (ELISA) kits (4A Biotech, Inc., Beijing, China) according to the manufacturer’s protocols.

### Quantitative Real-Time Polymerase Chain Reaction (qRT-PCR) Assay

The total RNA was extracted from infected brain tissues and RAW264.7 cells using Trizol reagent (Transgen Biotech, China) following the manufacturer’s protocols. Subsequently, the total RNA (1 μg) was reverse-transcribed to cDNA using a reverse transcription kit (TaKaRa, Japan). Gene-specific primers for quantitative real-time PCR was designed using Premier 7.0 software (Premier Biosoft International, Palo Alto, CA, United States), the gene-specific quantitative real-time PCR primes are listed in Table 1. Quantitative RT-PCR was performed on ABI Step one plus real-time PCR instrument using SYBR Green qPCR Master Mix (TaKaRa, Japan). The expression levels of target genes were normalized to β-tubulin levels using the 2-^ΔΔCt^ method.

**Table 1:** Primers used for qRT-PCR.

Name	Forward primer(5′–3′)	Reverse primer(5′–3 ′)
SAG1	TGCGATGTGGCGCTATGG	TTTTATCTGGGCAGGTGACAACT
BAG1	GACTGAGCGAGTGTCCGGTTA	TTCCGTCGGGCTTGTAATTACT
β-tubulin	CACTGGTACACGGGTGAAGGT	ATTCTCCCTCTTCCTCTGCG

### Intracellular Replication Assay

After RAW264.7 cells pretreated with etanercept (10, 100, 1000 ng/mL) for 1 h, freshly harvested tachyzoites were purified by filtration through 3 mm polycarbonate membranes was used to infect RAW264.7 cells inoculated on coverslips in 24-well plates at 37°C with 5% CO_2_ for 1 h. Then non-invaded tachyzoites were washed away by PBS, and the remaining ones continued to be cultivated for 24 h. The cultures were fixed with 4% paraformaldehyde and stained with swine anti-Toxoplasma IgG (to stain extracellular parasites). Then the samples were washed by PBS, subsequently were permeabilized with 0.1% Triton X-100 for 15 min and stained with rabbit anti-TgALD. After extensive washing, the samples were stained with secondary antibodies Alexa Fluor 594-conjugated goat anti-rabbit IgG and FITC conjugated goat anti-swine IgG, respectively (Life Technologies, Camarillo, CA, United States). Extracellular parasites that were stained both green and red were not counted. The number of parasites in each PV was counted three times independently.

### Statistical Analysis

All data were performed with GraphPad Prism 7 (GraphPad InStat Software, CA, United States) and expressed as the mean ± standard error of the mean (SEM) of three independent trials, and the statistical differences between the experimental groups were determined using the Student’s *t*-test or two-way analysis of variance as indicated in the figure legends. A *p*-value of < 0.05 was considered to be statistically significant.

## Results

### *In vivo* Study

#### Establishment of Chronic *T. gondii* Infection in Mice

These mice were randomly divided into seven groups (6 mice each group) to be infected with 10 freshly cysts of ME49 strain through intragastric administration. After 4 weeks, the levels of bradyzoite antigen 1 (BAG1) in the serum of mice were detected by a previously established ELISA method using the recombinant TgBAG1 protein (Doskaya et al., 2014). As shown in Table 2, a total of 30 mice were anti-BAG1 positive 4 weeks post-infection. Therefore, we successfully established a mouse model of chronic infection with *T. gondii*, and these mice were used for subsequent experiments.

**Table 2:** The BAG1 antibody level in the serum of mice infected with *T. gondii* cysts after 4 weeks.

	OD_630_ of serum samples from infected mice					
Groups	#1	#2	#3	#4	#5	#6
1st group	**0.588**	**0.565**	**0.639**	**0.462**	**0.586**	**0.422**
2nd group	**0.41**	0.339	**0.551**	**0.493**	**0.462**	**0.548**
3rd group	**0.808**	**0.611**	**0.518**	**0.429**	**0.466**	**0.625**
4th group	**0.704**	**0.408**	0.358	0.333	**0.396**	**0.386**
5th group	0.279	0.345	0.113	**0.396**	**0.49**	0.33
6th group	0.34	0.333	0.377	**0.383**	**0.386**	**0.503**
7th group	**0.546**	**0.453**	0.158	**0.389**	0.314	**0.588**

*Mice were orally infected with 10 freshly isolated cysts of the ME49 strain, antibody levels to the BAG1 protein in the serum of infected mice were examined by ELISA (means of three independent experiments). The cut-off value for positive calls is 0.38 and positive samples were indicated in bold.*

#### Etanercept Treatment Aggravates Clinical Symptoms and Increases Mortality in Mice Chronically Infected With *T. gondii*

**FIGURE 1:**
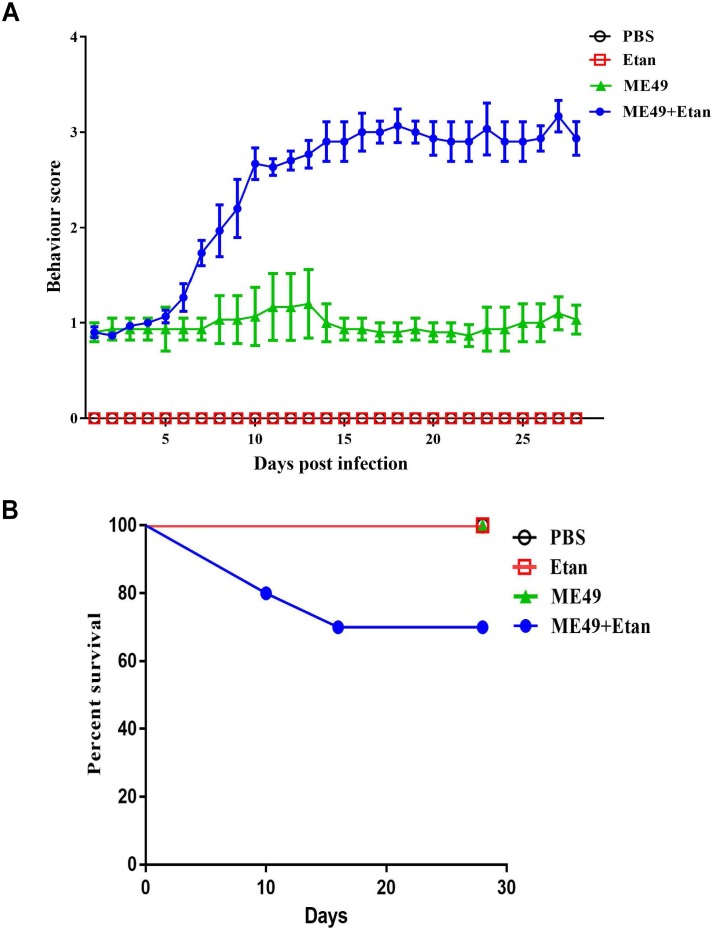
Effects of etanercept on clinical symptom and mortality of mice chronically infected with *T. gondii*. **(A)** Behavior score of mice. Note: Behavior score. 0 = no restriction of movement; no blink frequently; no body stiffening; no hind limb paralysis. 1 = no restriction of movement; blink frequently; no blink frequently; no body stiffening; no hind limb paralysis. 2 = restriction of movement; blink frequently; no body stiffening; no hind limb paralysis. 3 = restriction of movement; blink frequently; no hind limb paralysis. 4 = restriction of movement; eyes closed; body stiffening; hind limb paralysis; sometimes tremor. **(B)** Mortality of mice in each group (*n* = 10 for each group).

To explore the effects of etanercept on the survival of mice, we examined clinical symptoms and mortality in mice chronic infection with *T. gondii* for 30 days. As shown in Figure 1A, there was no clinical manifestation in PBS and Etan groups, demonstrating that etanercept had no toxic effects on mice. A worse behavior was observed in ME49+Etan group compared with ME49 group. Most of mice in ME49+Etan group behaved sluggish movement and frequent blinking, and some of them even showed symptoms of stiff body, these results suggested that etanercept treatment enhanced clinical symptoms caused by *T. gondii* infection. Four weeks post-observation, all mice in PBS, Etan and ME49 groups survived, and 30% mortality was measured in ME49+Etan group (Figure 1B). Compared with the ME49 group, the survival rate of the mice after treatment with etanercept was reduced, indicating that etanercept treatment could increase mortality of mice on the status of chronic infection.

#### Etanercept Treatment Exacerbates *T. gondii*-Induced Encephalitis

**FIGURE 2:**
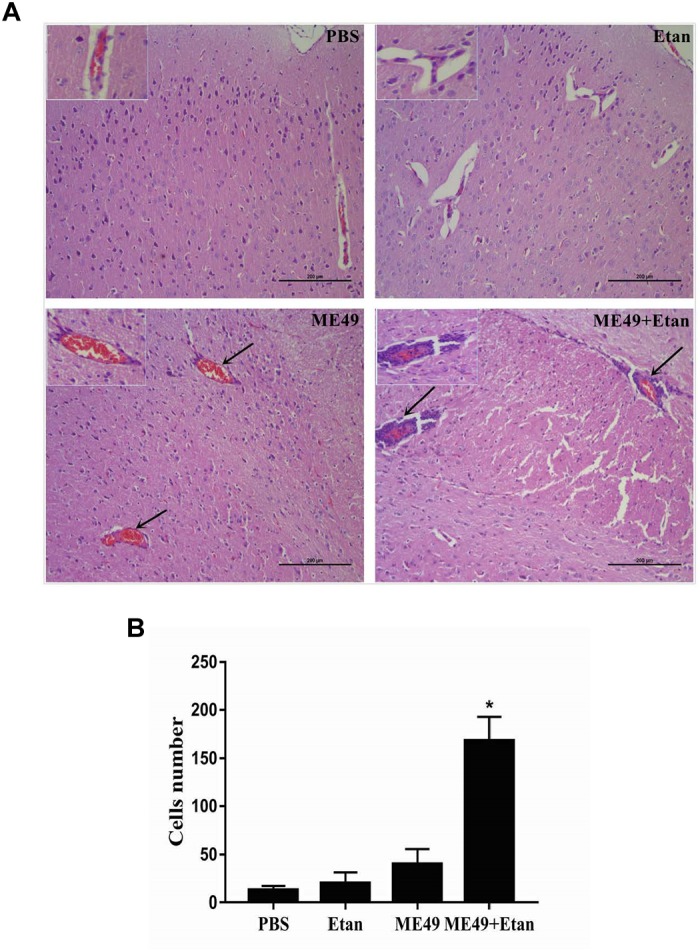
Effects of etanercept on the pathological changes of mice chronically infected with *T. gondii*. **(A)** Histopathological analysis of brain tissues. **(B)** The number of inflammatory cells around the blood vessels. PBS is the PBS treatment group; Etan is the etanercept treatment group; ME49 is the group that ME49 strain chronically infected with mice; ME49 + Etan is the group that ME49 strain plus etanercept treatment. The black arrows indicate the inflammatory cells infiltration around the blood vessels. The Data are presented as the mean ± SEM of three independent experiments. ^∗^*p* < 0.05 vs. ME49 group, student’s *t*-test.

The brain tissues in each group were harvested 4 weeks post-infection, and then brain histopathology was determined using H&E staining. There were no obvious pathological changes in PBS and Etan treatment groups. Mice in ME49 group showed a small amount of inflammatory cells and erythrocytes infiltration. However, etanercept treatment obviously exacerbated the development of *T. gondii*-induced encephalitis, which showed a large number of inflammatory cells infiltration around the blood vessels and destructed structure of the meninges (Figure 2A). Furthermore, the counting results suggested that inflammatory cells infiltration around the blood vessels in ME49+Etan group presented a significant increase compared with brain tissues sections in ME49 group (Figure 2B).

#### Etanercept Treatment Downregulates the Production of Cytokines in Serum

**FIGURE 3:**
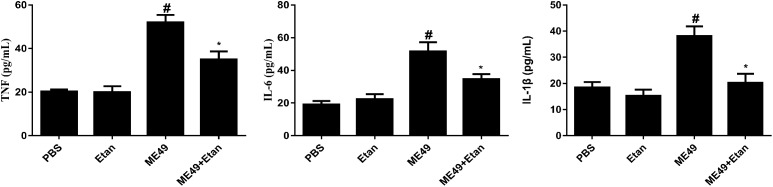
Effects of etanercept on cytokines production in serum. The expression levels of TNF, IL-1β, and IL-6 in serum was measured by ELISA. PBS is the PBS treatment group; Etan is the etanercept treatment group; ME49 is the group that ME49 strain chronically infected with mice; ME49 + Etan is the group that ME49 strain plus etanercept treatment. The data are presented as the mean ± SEM of three independent experiments. ^∗^*p* < 0.05 vs. ME49 group; ^#^*^p^* < 0.05 vs. PBS group, student’s *t*-test.

The effects of etanercept on the production of cytokines in serum was measured by ELISA assay, the results showed that the expression levels of TNF, IL-1β, and IL-6 were significantly increased after *T. gondii* stimulation compared with control groups. Nevertheless, etanercept significantly downregulated *T. gondii*-induced the increase of TNF, IL-1β, and IL-6 (Figure 3).

#### Etanercept Treatment Increases the Brain Cysts Number in Mice Chronically Infected With *T. gondii*

**FIGURE 4:**
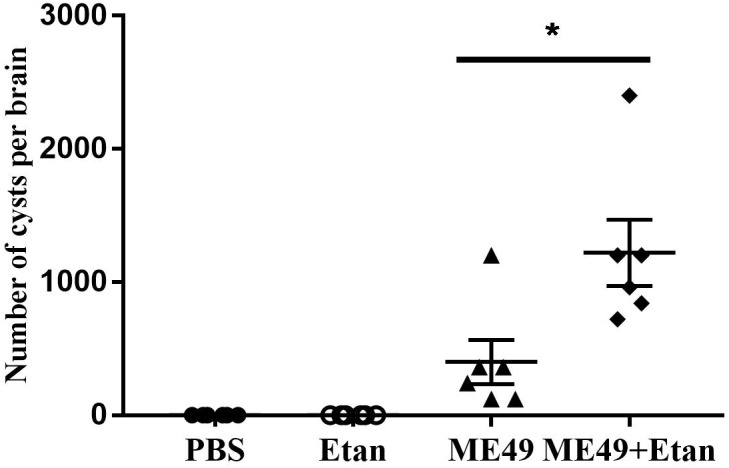
Effects of etanercept on the brain cysts number in mice chronically infected with *T. gondii*. The brain cysts were stained by DBA, then the brain cysts number was counted using fluorescence microscope. PBS is the PBS treatment group; Etan is the etanercept treatment group; ME49 is the group that ME49 strain chronically infected with mice; ME49 + Etan is the group that ME49 strain plus etanercept treatment. The data are presented as the mean ± SEM of three independent experiments. ^∗^*p* < 0.05 vs. ME49 group, student’s *t*-test.

In the present study, we explored the changes of brain cysts number after etanercept treatment using DBA staining, and then the brain cysts number was counted under fluorescence microscope. After etanercept treatment, the mean number of brain cysts showed a significant increasing trend compared with infected control group, while brain cysts were not observed in PBS and Etan groups (Figure 4). The results illuminated that etanercept treatment may increase the brain cysts number in mice.

#### Activation of Chronic *T. gondii* Infection in Mice After Etanercept Treatment

**FIGURE 5:**
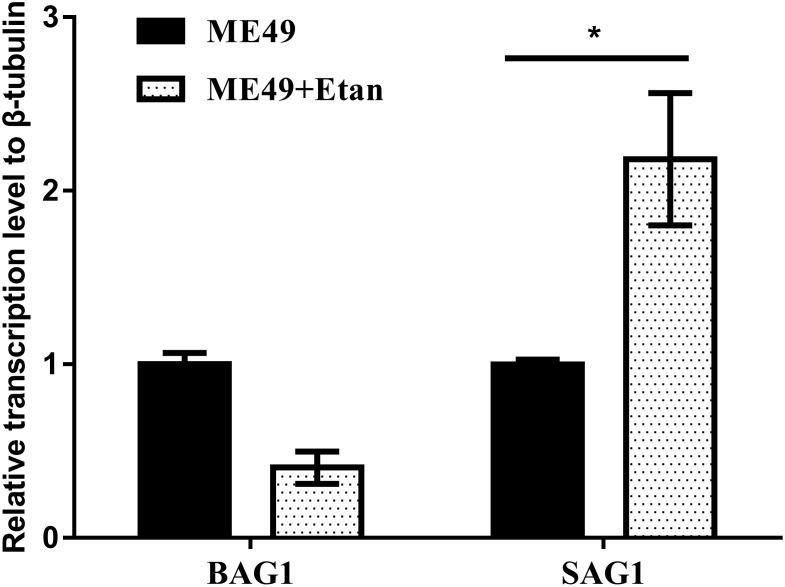
Effects of etanercept on the relative expression levels of BAG1 and SAG1 in mice brain chronically infected with *T. gondii*. The mRNA expression levels of BAG1 and SAG1 were measured by qRT-PCR. β-tubulin was used as internal control. ME49 is the group that ME49 strain chronically infected with mice; ME49 + Etan is the group that ME49 strain plus etanercept treatment. The data are presented as the mean ± SEM of three independent experiments. ^∗^*p* < 0.05 vs. ME49 group, student’s *t*-test.

Quantitative RT-PCR analysis was used to detect the expression levels of tachyzoite specific gene SAG1 and bradyzoite specific gene BAG1. As shown in Figure 5, the expression levels of SAG1 significantly increased, indicating that the brain cysts may reactivate from bradyzoites to tachyzoites with etanercept treatment.

### *In vitro* Study

#### Effect of Etanercept Treatment on Cell Viability

**FIGURE 6:**
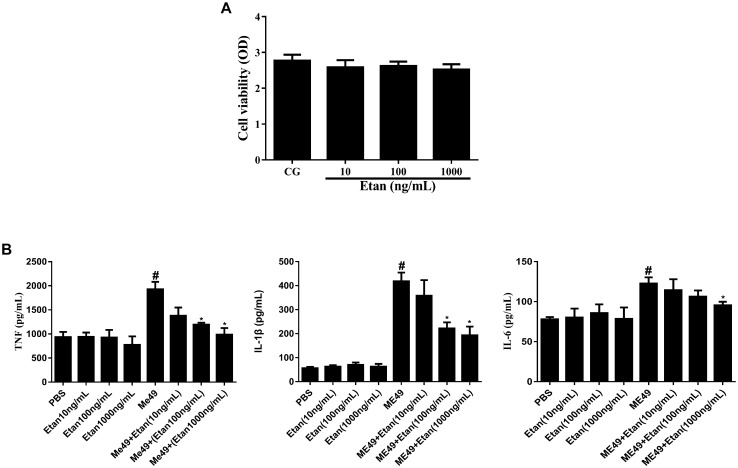
**(A)** Effects of etanercept on cell viability. RAW 264.7 cells were cultured with different concentrations of etanercept (10, 100, and 1000 ng/mL) for 24 h, and then the cell viability was measured using the MTT assay. **(B)** Effects of etanercept on cytokines production in RAW264.7 cells infected with *T. gondii*. The proteins expression levels of TNF, IL-1β, and IL-6 in cells were measured by ELISA. PBS is the PBS treatment group; Etan (10, 100, and 1000 ng/mL) are the etanercept (10, 100, and 1000 ng/mL) treatment groups; ME49 is the group that ME49 strain chronically infected with mice; ME49 + Etan (10, 100, and 1000 ng/mL) are the groups that ME49 strain plus etanercept (10, 100, and 1000 ng/mL) treatment. The data are presented as the mean ± SEM of three independent experiments. ^∗^*p* < 0.05 vs. ME49 group; ^#^*p* < 0.05 vs. PBS group, student’s *t*-test.

To assess if etanercept had any cytotoxicity on RAW264.7 cells, MTT assay was applied to measure the cell viability. Results in Figure 6A certified that there was no effect on cell viability caused by etanercept treatment.

### Etanercept Treatment Downregulates the Production of Cytokines in RAW264.7 Cells

The effects of etanercept on the production of cytokines in RAW264.7 cells were measured by ELISA assay. These results showed that the production of TNF, IL-1β, and IL-6 significantly increased after *T. gondii* infection. By contrast, etanercept treatment does dependently downregulated *T. gondii*-induced the increases of TNF, IL-1β, and IL-6, which were consistent with *in vivo* experiments (Figure 6B).

### Activation of Bradyzoites Infection in RAW264.7 Cells After Etanercept Treatment

**FIGURE 7:**
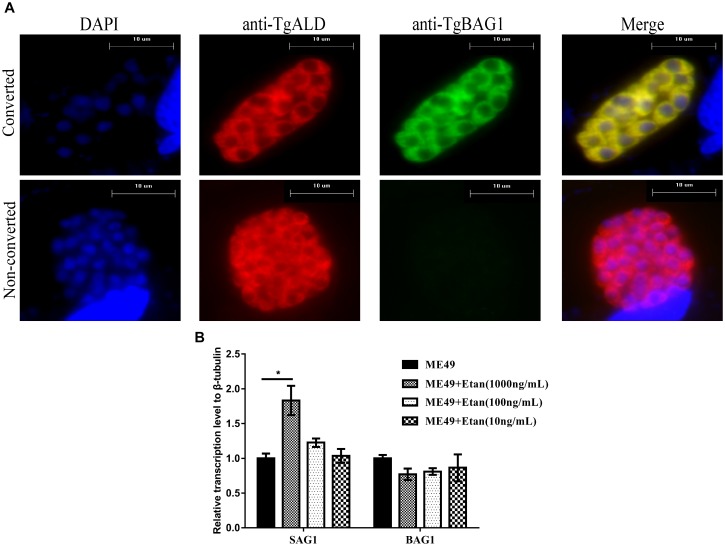
Effects of etanercept on the relative expression levels of BAG1 and SAG1 in RAW264.7 cells infected with bradyzoites. **(A)** Examples of parasites that were converted (BAG1^+^) or not converted (BAG1^−^) to bradyzoites. Samples were fixed and stained with mouse anti-BAG1 and goat anti-ALD, which were detected by Alexa 488- and Alexa 594-conjugated secondary antibodies, respectively. DAPI, images with emission filter: 440/40 nm; BAG1, images with emission filter: 530/30 nm; ALD, images with emission filter: 610/40 nm. Scale bars, 10 μm. **(B)** The mRNA expression levels of BAG1 and SAG1 were measured by qRT-PCR. β-tubulin was used as internal control. ME49 is the group that ME49 strain chronically infected with mice; ME49 + Etan (10, 100, and 1000 ng/mL) are the groups that ME49 strain plus etanercept (10, 100, and 1000 ng/mL) treatment. The data are presented as the mean ± SEM of three independent experiments. ^∗^*p* < 0.05 vs. ME49 group, student’s *t*-test.

To verify whether etanercept treatment also exerted the effect of reactivating from bradyzoites to tachyzoites *in vitro*, RAW264.7 cells were infected with bradyzoites. Before the parasites were inoculated to RAW264.7, ME49 tachyzoites were induced with alkaline conditions in HFF monolayers for 4 days. Almost all tachyzoites were converted to bradyzoites, as detected by positive staining for a bradyzoite marker BAG1 (Figure 7A). Subsequently, the expression levels of tachyzoite specific gene SAG1 and bradyzoite specific gene BAG1 were determined via quantitative RT-PCR analysis. The results in Figure 7B showed that only 1000 ng/mL of etanercept treatment could significantly increase the expression levels of SAG1. These results suggested that etanercept treatment could promote the transformation of bradyzoites to tachyzoites, however, only high-dose etanercept could take effect.

### Effect of Etanercept Treatment on Intracellular Replication Ability of Tachyzoites in RAW264.7 Cells

**FIGURE 8:**
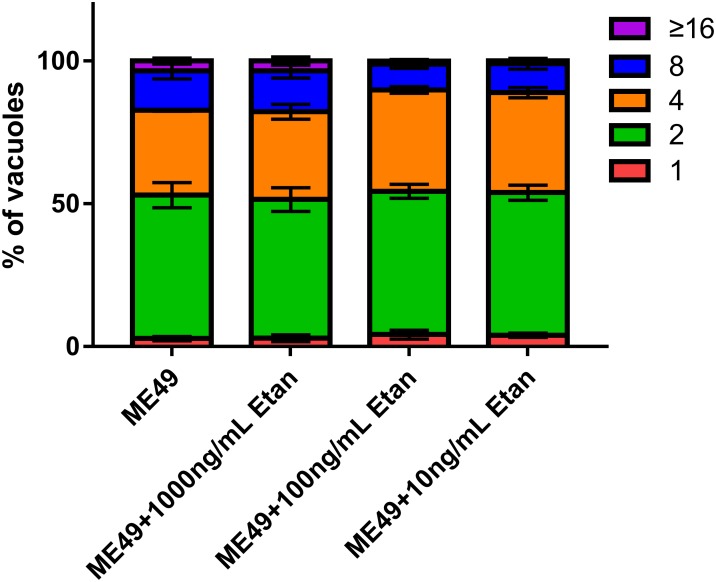
Effects of etanercept on the intracellular replication of tachyzoites in RAW264.7 cells. Purified tachyzoites were used to infect RAW 264.7 cells and invaded parasites were cultured for 24 h before fluorescent staining to determine the number of parasites in each parasitophorous vacuole (PV). PBS is the PBS treatment group; Etan (10, 100, and 1000 ng/mL) are the etanercept (10, 100, and 1000 ng/mL) treatment groups; ME49 is the group that ME49 strain chronically infected with mice; ME49 + Etan (10, 100, and 1000 ng/mL) are the groups that ME49 strain plus etanercept (10, 100, and 1000 ng/mL) treatment. The Data are presented as the mean ± SEM of three independent experiments, *p* > 0.05, two-way analysis of variance.

Tachyzoites are the main pathogenic forms of *T. gondii*, therefore, we assessed the replication of tachyzoites in RAW264.7 cells after etanercept treatment. As shown in Figure 8, three different concentrations of etanercept had no significant effect on the replication ability of tachyzoites in RAW264.7 cells, indicating that replication ability of tachyzoites was not regulated by etanercept *in vitro*.

## Discussion

As a worldwide distributed intracellular parasite, *T. gondii* is an important zoonotic pathogen (Lv et al., 2017). Toxoplasmosis often assumes chronic infection clinically, the brain cysts are persistent in host with no obvious pathological symptoms (Hunter et al., 1996). However, for host whose immunity is inhibited, the cysts can reactivate from bradyzoites to tachyzoites, which may cause lethal toxoplasmosis (Basavaraju, 2016). TNF is a cytokine involved in inflammation and immune response, and plays an important role in resisting *T. gondii* (Johnson, 1992; Lang et al., 2007). Etanercept is a competitive TNF inhibitor, and is widely used in clinical treatment to cure autoimmune diseases. It has been reported that peritoneal administration of etanercept also has effects on the reduction of neutrophil recruitment in IL-1β or LPS-induced brain tissue damage (Campbell et al., 2007). Recent research has indicated that etanercept treatment may be a risk of increasing the number and size of brain tissue cysts in latent toxoplasmosis (El-Sayed et al., 2016). In addition to the two cases previously reported, it was, similarly, found that two patients expressed positive and high levels of anti-*T. gondii* specific antibodies and developed toxoplasmic chorioretinitis during anti- TNF drugs (Gonzalez-Vicent et al., 2003; Young and McGwire, 2005; Lassoued et al., 2007). However, the specific molecular mechanisms of etanercept reactivating chronic toxoplasmosis are still unknown. In the present study, a mouse model of chronic infection with *T. gondii* was successfully established, and we investigated whether etanercept reactivates latent toxoplasmosis and the specific mechanisms of this phenomenon.

In our study, compared with other three groups, the survival rate of mice treated with etanercept reduced to 70%, and the mental state was significantly worse. Furthermore, the histological results showed that *T. gondii* could cause severe brain pathological changes, which would be aggravated by etanercept treatment. These results indicated that etanercept may exacerbate the clinical pathological symptoms induced by *T. gondii*.

It has been reported that the use of anti-TNF antibodies against mice or macrophages can induce the transformation of bradyzoites to tachyzoites and tachyzoites proliferate (Rodrigues et al., 2013). Therefore, we detected relative expression levels of BAG1 and SAG1 mRNA in mice brain. From the results, it can be seen that the expression levels of SAG1 in the infected mice treated with etanercept was significantly increased, while the expression levels of BAG1 was decreased but not significant. Combined with the results of brain cysts number, brain cysts number in mice treated with etanercept was significantly increased, suggesting that etanercept may promote the transformation of bradyzoites to tachyzoites, tachyzoites rapidly proliferated, and then form new brain cysts in host brain tissues.

Recent research has shown that treated with IL-1β alone or in combination with TNF can significantly increase the survival rate in mice infected with *T. gondii*. And that, IL-1β can directly activate T lymphocytes and macrophages, promoting the production of IFN-γ (Chang et al., 1990). IL-6 can activate the cytotoxicity of eosinophils to *T. gondii*, and the production of IL-6 can induce the secretion of specific proteins during acute infection, thereby inhibiting the replication of parasites in the host (Chang et al., 1990; Koo et al., 2011). In our study, the expression levels of TNF, IL-1β, and IL-6 were decreased and the number of cysts was increased in mice chronically infected with *T. gondii* after being treated with etanercept. The decreases of these cytokines in host undergoing etanercept treatment may suggest reduced immune functions in host chronically infected with *T. gondii*. Reduced immune function in host activates the brain cysts, which would lead to conversion from chronic infection to acute infection, causing severe pathological symptoms. The above result may indicate that it could fatal to use etanercept in patients co-infected with chronic toxoplasma and autoimmune diseases, such as ankylosing spondylitis, psoriasis. Therefore, it is necessary to perform Toxoplasma serological testing in patients before treatment with etanercept and other biological anti-TNF agents, in addition, patients who have been treated with these drugs should be recommended to avoid the risk of infection with *T. gondii*. In addition, co-infection patients should be treated with classic sulfonamide therapy when they were accepted with etanercept and other biological anti-TNF agents, which could reduce the lesion size and inflammation (Soheilian et al., 2011).

It is well-known that macrophages can take regulatory effects between host and pathogens through releasing inflammatory cytokines and chemokines (Lim et al., 2015; Yang S. et al., 2017), and murine macrophages RAW264.7 have been widely employed to study the interaction between host immunity and parasites (Luder et al., 2003; Li et al., 2018). In the study, murine macrophages RAW264.7 cells were pretreated with etanercept, and then infected with *T. gondii*. The expression levels of cytokines and SAG1 were consistent with the results of *in vivo* experiments.

The rapidly replicated tachyzoites disseminates throughout the host during acute infection, which may cause serious clinical symptoms (Pittman and Knoll, 2015). Since we have determined that etanercept could increase the mortality of mice chronic infection with *T. gondii*, therefore, we examined whether etanercept had an effects on the intracellular replication ability of tachyzoites *in vitro*. From the results, it can be confirmed that different concentrations of etanercept have no effects on the intracellular replication ability of tachyzoites, suggesting that etanercept may only caused a decrease in the immune levels of the mice and activate the brain cysts without directly affecting the intracellular replication ability of tachyzoites. This phenomenon may be related to the fact that using TNF alone does not significantly affect intracellular *T. gondii* number (Adams et al., 1990). However, the specific mechanism of the effects of etanercept on the replication of tachyzoites is still not clear, needing further exploration.

## Conclusion

Etanercept treatment promoted the development of chronic toxoplasmosis by reducing the host cell immunity levels and promoting the transformation of bradyzoites to tachyzoites *in vivo* and *in vitro*. These studies are expected to be helpful for patients who are co-infected with autoimmune diseases and latent toxoplasmosis treated with this drug.

## Author Contributions

RF and JY conceived and designed the study, JY drafted the manuscript. JY, LW, DX, DT, SL, FD, LW, and JZ performed the experiments and analyzed the data. All authors read and approved the final manuscript.

## Conflict of Interest Statement

The authors declare that the research was conducted in the absence of any commercial or financial relationships that could be construed as a potential conflict of interest.
